# Anästhesiologisches und postinterventionelles Management bei perkutaner hepatischer Melphalanperfusion (Chemosaturation)

**DOI:** 10.1007/s00101-022-01235-3

**Published:** 2022-12-07

**Authors:** Peter Kliem, Sebastian Ebel, Robert Werdehausen, Felix Girrbach, Denis Bösemann, Florian van Bömmel, Timm Denecke, Sebastian Stehr, Manuel F. Struck

**Affiliations:** 1 Klinik und Poliklinik für Anästhesiologie und Intensivtherapie, Universitätsklinikum Leipzig, Liebigstr. 20, 04103 Leipzig, Deutschland; 2 Klinik und Poliklinik für Diagnostische und Interventionelle Radiologie, Universitätsklinikum Leipzig, Leipzig, Deutschland; 3 Klinik für Herz- und Thoraxchirurgie, Kardiotechnik, Universitätsklinikum Jena, Jena, Deutschland; 4 Klinik und Poliklinik für Onkologie, Gastroenterologie, Hepatologie, Pneumologie und Infektiologie, Bereich Hepatologie, Universitätsklinikum Leipzig, Leipzig, Deutschland

**Keywords:** Lebertumoren, Melphalan, Angiographie, Chemosaturation, Extrakorporalkreislauf, Liver tumors, Melphalan, Angiography, Chemosaturation, Extracorporeal circulation

## Abstract

Die perkutane hepatische Perfusion mit dem Chemotherapeutikum Melphalan (PHMP) ist eine Letztlinientherapie bei Patienten mit inoperablen primären oder sekundären Lebertumoren. Dabei wird die Leber transarteriell mit Melphalan perfundiert und aufgesättigt (Chemosaturation), mit dem Ziel, die Lebertumoren selektiv und ohne dessen systemische zytotoxische Eigenschaften zu behandeln. Über einen Extrakorporalkreislauf und eine Ballonokklusion der V. cava inferior wird das venöse hepatische Blut hämofiltriert und venös zurückgeleitet. Verfahrensbedingt kommt es dabei zu einer ausgeprägten Kreislaufdepression und einer Störung der plasmatischen Gerinnung. In diesem Artikel wird das anästhesiologische und postinterventionelle Management bei Patienten mit PHMP beschrieben und auf Fallstricke und Besonderheiten hingewiesen.

## Abstract (en)

Percutaneous hepatic melphalan perfusion (PHMP) is a last-line treatment of inoperable primary or secondary liver tumors. Selective perfusion and saturation (chemosaturation) of the liver with the chemotherapeutic agent melphalan is performed via catheterization of the hepatic artery without affecting the rest of the body with its cytotoxic properties. Using an extracorporeal circulation and balloon occlusion of the inferior vena cava, the venous hepatic blood is filtered and returned using a bypass procedure. During the procedure, considerable circulatory depression and coagulopathy are frequent. The purpose of this article is to review the anesthesiological and postprocedural management of patients undergoing PHMP with consideration of the pitfalls and special circumstances.

## Einleitung

Inoperable primäre oder sekundäre Lebertumoren sind Tumorerkrankungen mit insgesamt ungünstiger Prognose [28, 39, 44]. Das Aderhautmelanom weist dabei ein besonders aggressives hepatisches Metastasierungsverhalten auf [3, 54]. Obwohl die Einführung der Antikörpertherapie vielversprechende Ergebnisse insbesondere bei der Behandlung von metastasierenden kutanen Melanomen zeigt, können diese bei metastasierten Aderhautmelanomen nicht bestätigt werden [53]. Eine systemische Chemotherapie ist für hepatisch metastasierte Aderhautmelanome nicht etabliert.

Die selektive perkutane hepatische Perfusion mit dem Chemotherapeutikum Melphalan (Chemosaturation) ist ein Ansatz zur Tumorlastreduktion bzw. Tumorkontrolle, der insbesondere beim hepatisch metastasierten Aderhautmelanom mit einer guten Ansprechrate einhergeht [3, 7, 9, 14–16, 22, 23, 32, 35, 37, 39, 49]. Auch bei anderen Tumorentitäten, z. B. bei inoperablen hepatischen Metastasen von kutanen Melanomen, des kolorektalen Karzinoms, von neuroendokrinen Tumoren und bei Patienten mit hepatozellulärem Karzinom (HCC) oder intrahepatischem Cholangiokarzinom (ICC), kann häufig eine relevante Reduktion der Tumormasse erreicht werden [11, 14, 25, 30, 40, 46, 48, 50].

Bereits in den 1960er-Jahren wurden mit Untersuchungen zur Gefäßversorgung von Lebertumoren die Grundlage der selektiven Leberperfusion gelegt [8]. Dabei konnte erstmals gezeigt werden, dass die meisten primären und sekundären Lebertumoren hauptsächlich durch die *hepatischen Arterien* und deren Äste perfundiert werden, während gesunde Hepatozyten zu ca. 70 % durch die Pfortader versorgt werden [12, 45]. Diese Erkenntnis führte im weiteren Verlauf zu Therapiekonzepten von bis dato inoperablen Lebertumoren durch eine selektive isolierte hepatische Perfusion (IHP) mit hochdosierten Chemotherapeutika. Hierbei wurde offen chirurgisch eine komplexe Isolierung des Leberkreislaufs vom Körperkreislauf vorgenommen und anschließend das Chemotherapeutikum über eine Herz-Lungen-Maschine zugeführt [6]. Aufgrund des Komplikationspotenzials dieses Verfahrens [17] folgten in den 1990er-Jahren erste Schritte zu einer minimal-invasiven perkutanen hepatischen Melphalanperfusion (PHMP) [5]. Im Vergleich zur IHP zeigte die PHMP deutlich geringere Komplikationsraten und eine geringere 30-Tage-Sterblichkeit [7].

Es konnte mittlerweile in zahlreichen Studien gezeigt werden, dass die PHMP eine Behandlungsoption bei Patienten mit inoperablen, isolierten Lebermetastasen oder primären Lebertumoren darstellt [3, 9, 11, 14–17, 19, 22, 25, 29, 30, 33, 37, 38]. Allerdings ist die Auswahl geeigneter Patienten für eine PHMP entscheidend, um einen möglichst sicheren peri- und postoperativen Verlauf zu gewährleisten. Unerwünschte Wirkungen sind in diesem speziellen Setting durch die Interventionsschritte und die Toxizität des verwendeten Melphalans relativ häufig. Dabei spielen hauptsächlich vorübergehende Hypotension, Tachykardie und Koagulopathie eine Rolle; deren Management soll mit diesem Artikel etwas näher beleuchtet werden.

## Verfahren/Methode

Derzeit steht mit dem *Hepatic CHEMOSAT® Delivery System* (Fa. Delcath Systems Inc., New York, NY, USA) ein minimal-invasives Verfahren zur Behandlung von Lebertumoren zur Verfügung. Seit 2011 besteht in der Europäischen Union eine CE-Zulassung [11].

**Figure 1:**
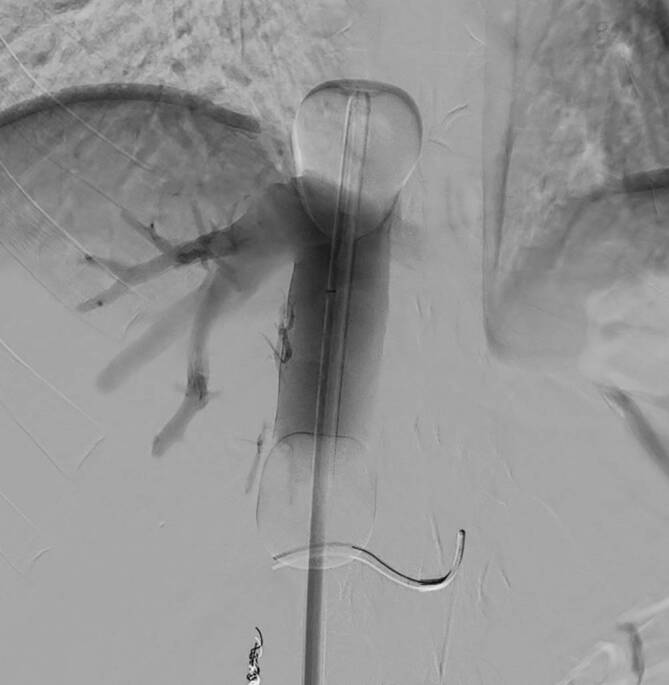

Dabei wird hochdosiertes Melphalan (3 mg/kg ideales Körpergewicht) durch ein interventionell eingebrachtes Kathetersystem direkt in die *hepatischen Arterien* injiziert. Dieses System wird über eine zuvor platzierte Schleuse in einer *A. femoralis communis* eingebracht. Dadurch werden die Tumorzellen in der Leber mit dem Chemotherapeutikum aufgesättigt. Um die systemische toxische Wirkung des Melphalans zu verhindern, wird über eine zweite Schleuse in der *V. femoralis* ein fenestrierter Doppelballonkatheter in der *V. cava* inferior positioniert (Abb. 1).

**Figure 2:**
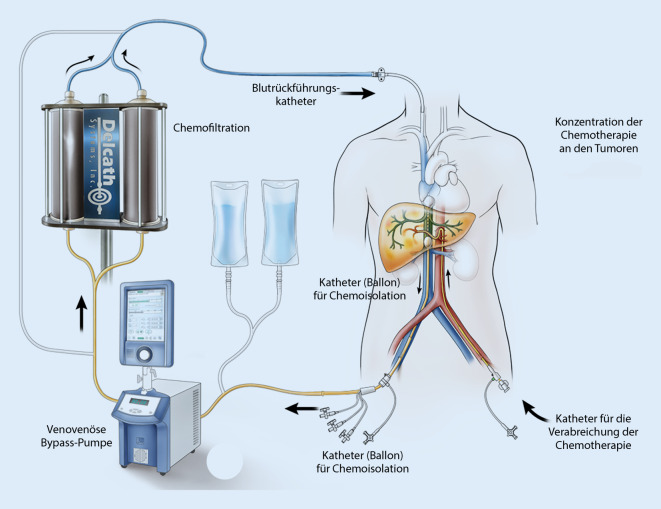

Auf diese Weise von der systemischen Zirkulation isoliert, kann das mit Melphalan aufgesättigte abfließende venöse Blut abgeleitet werden. Mittels einer extrakorporalen Zentrifugalpumpe wird das abfließende Blut durch spezielle Filter geführt und das Chemotherapeutikum zu ca. 95 % filtriert. Über eine weitere Schleuse in der rechten *V. jugularis interna* wird das gereinigte Blut in den Körperkreislauf zurückgeführt (Abb. 2). Der Einsatz dieses Pumpen‑/Filtersystems macht eine Vollheparinisierung des Patienten mit 300–400 IE Heparin/kgKG erforderlich, welche mittels *Activated Clotting Time* (ACT) gesteuert wird (Ziel: > 400 s). Die Steuerung und Überwachung der Extrakorporalpumpe werden durch einen Kardiotechniker durchgeführt. Die Heparinwirkung wird durch regelmäßige Bestimmungen der ACT überwacht. Nach der Beendigung der Melphalaninfusion wird eine halbstündige Auswaschphase durchgeführt, in der die Pumpe weiterläuft und schließlich abgekoppelt wird.

Dieses spezielle Verfahren mit chemotherapeutikumbindenden Filtern ermöglicht die Verabreichung höherer Wirkdosen von Melphalan, ohne schwere toxische Nebenwirkungen, die eine systemische Therapie mit sich bringen würde [27, 29, 30, 43]. Die Chemosaturation kann als Einzeleingriff oder wiederholt, abhängig vom klinischen Zustand und des Ansprechens des Tumors auf die Therapie, durchgeführt werden [3, 15, 47].

## Komplikationen

Die Chemosaturation ist ein Verfahren mit einem relevanten Komplikationsrisiko. Die Klassifikation der unerwünschten Ereignisse erfolgt dabei anhand der *Common Terminology Criteria for Adverse Events* (CTCAE) [36]. In den Studien zu Wirkung und Nebenwirkungen der Chemosaturation waren die häufigsten Komplikationen den Graden I (mild) und II (moderat) zuzuordnen, und Komplikationen mit den Graden III (schwerwiegend) und IV (lebensbedrohlich) traten selten auf [23, 35, 40, 43, 47]. Frühkomplikationen waren dabei periinterventionelle hämodynamische Komplikationen, die sowohl ausgeprägte Hypotonien als auch hypertensive Entgleisungen (meist überschießende Effekte im Rahmen einer Vasopressortherapie) einschlossen [40, 43]. Einblutungen an den Punktionsstellen waren z. T. mit raumfordernden Hämatomen verbunden [23, 30, 33, 40, 43, 47–50]. Dabei wurden auch vereinzelte Fälle mit Atemwegsobstruktion, erforderlicher Reintubation und schwierigem Atemweg beschrieben [43]. Selten wurde über schwere thrombembolische Komplikationen (Apoplex, Myokardinfarkt und Lungenarterienembolie) berichtet, die auch im Zusammenhang mit postinterventionellen Protamingaben standen [13, 22, 23, 32, 35, 40]. Zu den häufigsten frühen Spätkomplikationen wurden hämatotoxische Nebenwirkungen (Thrombopenie, Anämie und Leukopenie) beschrieben, welche bei fast allen Patienten auftraten [3, 11, 13–17, 19, 23, 25, 35, 37, 40, 43, 47–50]. Ebenfalls häufig beschrieben wurden passagere Anstiege der Transaminasen sowie eine Bilirubinerhöhung. In Einzelfällen trat ein Leberversagen auf [40]. Weitere unerwünschte Ereignisse waren Übelkeit und Erbrechen, Fieber, Troponinerhöhungen, Nachblutungen, Störungen des Elektrolythaushalts und Nephrotoxizität mit passagerer Dialysepflichtigkeit [2, 35, 40, 43]. Todesfälle wurden bei Patienten mit einer sehr hohen Tumorlast im Zusammenhang mit der Chemosaturation beschrieben [30, 40].

## Anästhesiologisches Management

### Aufklärung und Vorbereitung

Der Eingriff wird unter Allgemeinanästhesie mit endotrachealer Intubation durchgeführt. Neben der Aufklärung für eine Allgemeinanästhesie mit invasiver Blutdrucküberwachung sind präoperative Überlegungen zu Ort und Durchführung der notwendigen arteriellen und venösen Kanülierungen notwendig. Intraoperativ muss aufgrund der Okklusion der *V. cava inferior* und der Nutzung eines venovenösen extrakorporalen Bypasses mit einer ausgeprägten hämodynamischen Instabilität gerechnet werden. Die Okklusion führt, ähnlich wie beim Ausklemmen der *V. cava inferior* bei Leberteilresektionen oder Lebertransplantationen, zu einer akuten Vorlastsenkung mit schwerer Hypotonie [34, 42]. Daher sollte im Rahmen der Patientenevaluation ein besonderes Augenmerk insbesondere auf die kardiale Belastbarkeit der Patienten gelegt werden. Diesbezüglich gibt es derzeit keine klaren Kontraindikationen, wobei die Durchführung einer transthorakalen Echokardiographie zur Abschätzung der Pumpfunktion und des Vorhandenseins von Vitien als sinnvoll eingeschätzt wird. Patienten mit fortgeschrittener Leberzirrhose, vorbestehenden ausgeprägten Blutgerinnungsstörungen, Kontrastmittelallergie oder Vorliegen von extrahepatischen Metastasen, v. a. zerebralen Metastasen und Läsionen, sind aufgrund ihres Risikoprofils nicht für eine PHMP geeignet (Tab. 1; [13]).

**Table 1:** 

Kontraindikationen für eine Chemosaturationsbehandlung
Leberinsuffizienz oder portale Hypertension
Intrazerebrale Metastasen und andere extrahepatische Tumormanifestationen, die die Gesamtprognose relevant beeinflussen
Intrazerebrale Läsionen mit Blutungsgefahr
Allergien gegen Kontrastmittel, Heparin, Latex oder Melphalan
Z. n. chirurgischer oder interventioneller Leberbehandlung in den letzten 4 Wochen
Z. n. Kausch-Whipple-Operation oder pyloruserhaltender Pankreaskopfresektion (relativ, Einzelfallentscheidung, bei stabilem Allgemeinzustand PHMP möglich)
Tumorlast der Leber > 50 % (relativ, Einzelfallentscheidung, häufig schlechtes Ansprechen auf PHMP)
Gerinnungsstörungen, die mit einer relevant erhöhten Blutungsneigung einhergehen oder allgemeine Kontraindikationen für eine Vollheparinisierung

*PHMP* Perkutane hepatische Melphalanperfusion

Die Patienten sollten neben den bekannten Risiken einer Allgemeinanästhesie explizit auch über das Risiko schwerwiegender Komplikationen aufgeklärt werden. Dies sind insbesondere allergische Reaktionen auf Melphalan und weitere Pharmaka. Hinzu kommen das Risiko für Blutungskomplikationen im Rahmen der notwendigen Antikoagulation, aber auch thrombembolische Komplikationen (Apoplex, Myokardinfarkt und Lungenarterienembolie) als mögliche Folge der Protamingabe. Als weitere Risiken sind ein Herz-Kreislauf-Versagen, die Notwendigkeit einer Nachbeatmung oder erneuten Intubation, ein Nieren‑, Leber- oder Multiorganversagen mit der Notwendigkeit zum Einsatz von Organersatzverfahren (insbesondere Hämodialyse) sowie eine hierdurch verlängerte Intensivtherapie zu benennen. Präoperativ werden in unserem Zentrum jeweils 4 Erythrozytenkonzentrate und gefrorene Frischplasmakonzentrate sowie 2 Thrombozytenkonzentrate eingekreuzt.

### Anästhesiologisches Management

Der Arbeitsplatz in der Angiographieeinheit ist häufig in einem Außenbereich lokalisiert, sodass die Ressourcen eines zentralen Operationsbereichs nicht oder nur eingeschränkt zur Verfügung stehen.

Die Allgemeinanästhesie kann sowohl als balancierte Anästhesie oder als totale intravenenöse Anästhesie (TIVA) mit kontinuierlicher oder diskontinuierlicher Applikation von Opioiden erfolgen [43]. Nach Präoxygenation, Laryngoskopie, Intubation und Lagekontrolle erfolgt die sorgfältige Fixierung des Tubus. Die Anästhesietiefe sollte adäquat sein, da intraoperativ eine Dislokation der platzierten Okklusionsballons vermieden werden muss. Dabei kann die Verwendung der Relaxometrie als neuromuskuläres Monitoring hilfreich sein. Leckagen an den beiden Okklusionsballons könnten zum Entweichen des Chemotherapeutikums in den systemischen Kreislauf und entsprechenden toxischen Wirkungen vom Melphalan führen (Übelkeit, Erbrechen, Diarrhö, Alopezie, Stomatitis, Infertilität, Niereninsuffizienz, Leberinsuffizienz, Knochenmarkdepression). Daher erfordert die sichere Platzierung der Ballons große Aufmerksamkeit [13, 35, 40].

Nach der 3‑ bis 4‑stündigen Interventionsdauer kann die Allgemeinanästhesie im Regelfall ohne Verzögerung ausgeleitet werden (Tab. 2).

**Table 2:** 

*Anästhesieeinleitung und Katheterplatzierungen*	Standardmonitoring, Anlage einer peripheren Venenverweilkanüle
	Einleitung der Allgemeinanästhesie (Intubationsnarkose)
	Anlage des Harnblasenkatheters mit intravesikaler Temperaturmessung
	Anlage der arteriellen Blutdruckmessung (*A. radialis*)
	Sonographie, Übersicht, Hals (Halsgefäße bds., gemeinsam mit Radiologie)
	Anlage eines ZVK (mit mindestens einem großen Lumen) + venöse Schleuse des Extrakorporalsystems als Doppelpunktion, bevorzugt in die *V. jugularis interna dextra* (zwingend sonographiegestützt)
	Anlage der arteriellen und venösen Schleuse durch die Radiologie (*A. und V. femoralis communis*)
	Heparingabe **nach** Anlage aller venösen und arteriellen Zugänge mit 300 IE/kgKG, Ziel Activated Clotting Time (ACT) > 400 s
	Platzierung des Chemosaturationssystems (arterieller Katheter und venöser Doppelballonkatheter) durch Radiologie
*Anschluss des Extrakorporalsystems*	Vor Anschluss des Extrakorporalsystems **muss** die ACT > 400 s sein, ggf. Heparinnachdosierung und erneute ACT-Kontrolle
	Laufende Noradrenalinspritzenpumpe, mittlerer arterieller Druck (MAD) 80–90 mm Hg
	Start des Extrakorporalsystems durch Kardiotechnik
*Inflation der Ballons*	*Cave*: Blutdruckabfall zu erwarten, Bypass noch offen
	Lage- und Leckagekontrolle der Ballons durch Radiologie (bei Leckage Gefahr systemischer Wirkung des Chemotherapeutikums)
*Abklemmen des Bypass*	Kontrolle ACT, ggf. erneute Heparingabe
	Vor Abklemmen des Bypass Ziel-MAD 80–90 mm Hg
	*Cave*: deutlicher Blutdruckabfall (hohe Noradrenalindosis erforderlich)
	Mit dem Verschließen des Bypass Gabe von Volumenbolus (z. B. 500 ml balancierte Vollelektrolytlösung)
	Nach Stabilisierung MAD, Freigabe für Injektion der Chemotherapie in die Leberarterie durch Radiologie (Melphalan 3 mg/kgKG, max. 220 mg/Sitzung)
*Lokale Applikation des Chemotherapeutikums*	*Cave*: Bypass muss vor Applikation des Chemotherapeutikums geschlossen sein
	Ca. 30 min Applikationsdauer des Chemotherapeutikums
	Danach 30 min Auswaschphase mit Extrakorporalsystem
	Währenddessen ACT-Kontrolle
*Beendigung der Chemosaturation*	Nach Beendigung des Extrakorporalsystems Anstieg des MAD mit deutlicher Reduzierung der Noradrenalindosis zu erwarten
	Entfernung arterieller Katheter und venöser Doppelballonkatheter aus den Schleusen
	Alle Schleusen und ZVK werden in situ belassen
	Nach Auswaschphase im Konsens mit Radiologie Antagonisierung des Heparins durch Protamin (Protamindosis entsprechend der verabreichten Heparindosis) und danach Abschluss-ACT (angestrebt < 150 s)
	Sonographie, Übersicht, Hals (Ausschluss Blutungen/Ödeme, gemeinsam mit Radiologie)
	Beendigung der Anästhesie und Extubation (auf Normothermie achten)
	Verlegung auf die Intensivstation
*Postoperatives Management*	Aufnahmelabor mit Blutbild, Elektrolyte, Leber- und Nierenwerte und Gerinnungsparameter. Regelmäßige Verlaufskontrollen
	Entfernung aller zentralen Zugänge und arterielle Blutdruckmessung nach Normalisierung der Gerinnung (in der Regel am Folgetag). Ggf. Substitution. Druckverband auf Punktionsstelle der arteriellen Schleuse für 12 h
	Verlegung auf die Normalstation am Folgetag bei komplikationslosem Verlauf
	Gabe von Allopurinol zur Prophylaxe eines Tumorlysesyndroms

*ACT* „Activated clotting time“, *MAD* mittlerer arterieller Druck

### Gefäßzugänge

Nach Einleitung der Anästhesie, endotrachealer Intubation und Beginn der maschinellen Beatmung werden in unserem Zentrum 2 zentrale Venenkatheter (ZVK) in die *V. jugularis interna dextra* platziert. Ein ZVK sollte für die Gabe von erforderlichen Volumenboli mit einem High-Flow-Lumen (12 G) ausgestattet sein. Es können alternativ auch Dialysekatheter mit zusätzlichem kleinlumigen Schenkel für die Vasopressorinfusion verwendet werden, insbesondere, wenn absehbar ist, dass postinterventionell eine Nierenersatztherapie notwendig ist. Der zweite Zugang ist die im Hersteller-Set enthaltene venöse Schleuse zur Rückführung des filtrierten Blutes (10-F-Schleuse des *Hepatic CHEMOSAT® Delivery System*). Die arterielle Blutdruckmessung kann über einen separaten Zugang einer *A. radialis* etabliert werden oder über die Schleuse des Interventionssystems an der *A. femoralis* abgeleitet werden. Die Platzierungen der arteriellen und venösen Femoralschleusen für das Chemosaturationssystem werden durch den Interventionsradiologen durchgeführt.

Im Rahmen eigener Erfahrungen kam es in einigen Fällen zu mechanischen Komplikationen bei den Gefäßpunktionen, wobei diese meistens geringfügig waren (z. B. nichtvorschiebbarer Führungsdraht) [43]. Nach Mehrfachpunktionen kam es in einem Fall bereits vor der interventionsbedingten Heparinisierung zu einem ausgeprägten zervikalen Hämatom, sodass die Intervention aufgrund der Gefahr der Progression des Hämatoms bei Heparinisierung abgebrochen werden musste. Arterielle Blutungen im Bereich der Leistenregion und des Oberschenkels wurden nach Dislokation einer Schleuse beschrieben [43]. Um das Risiko von punktionsbedingten Blutungen insbesondere unter der intra-interventionell ausgeprägten Heparinisierung zu minimieren, sollten folgende Punkte beachtet werden:

Die Punktionen der zentralen Zugänge (ZVK und venöse Rückgabeschleuse) sollen primär unter ultraschallgeführter ständiger Nadelspitzenvisualisierung („real-time ultrasound guidance“) erfolgen, um die Fehlpunktionsrate bzw. die Mehrfachpunktionsrate zu verringern und das Blutungsrisiko bei der erforderlichen hochdosierten Heparingabe zu vermindern [2, 27]. Die primäre Punktionsstelle ist die rechte *V. jugularis interna*. Die Doppelpunktion der *V. jugularis interna dextra* ist erfahrungsgemäß problemlos möglich und bietet durch den anatomischen Gefäßverlauf Vorteile gegenüber der *V. jugularis interna sinistra*, bei deren Katheterisierung auf eine adäquate Einführtiefe und Spitzenposition im Bereich des kavoatrialen Überganges geachtet werden sollte [4, 42, 51, 52]. Bei Notwendigkeit einer Punktion der linken *V. jugularis interna* sollten der längere intravaskuläre Verlauf und das mögliche Risiko von Intimaläsionen bei zu kurzer Einführtiefe des ZVK berücksichtigt werden [20]. Grundsätzlich empfiehlt es sich, vor dem sterilen Abdecken der ausgewählten Punktionsstelle eine Übersichtssonographie von rechter und linker *V. jugularis interna* durchzuführen, um Kalibergrößen und benachbarte Arterien einzuschätzen [41]. Die Vermeidung der Punktion der *V. subclavia* ist angesichts des Risikos von Punktionsläsionen (Pneumothorax und intrathorakale Blutungen) empfehlenswert. Eine Lagekontrolle des ZVK kann neben dem endovaskulären EKG (bei Patienten ohne Vorhofflimmern) auch angiographisch unter Durchleuchtung stattfinden [18]. Zur Vermeidung von Dislokationen sind eine Annaht der Katheter und ein sorgfältiger Verband erforderlich. Die Punktionsstellen sollten während der Intervention regelmäßig auf eine Hämatomentwicklung und Blutungen kontrolliert werden [1, 2]. Nach der Auswaschphase, noch vor der Ausleitung der Anästhesie, ist eine erneute Übersichtssonographie des Halses sinnvoll, um Blutungen und Ödeme auszuschließen, die möglicherweise einer Extubation im Angiographiebereich entgegenstehen und eine spätere Extubation auf der Intensivstation erforderlich machen können.

### Gerinnungsmanagement

Vor Etablierung des Extrakorporalkreislaufs werden 300–400 IE Heparin/kgKG i.v. appliziert (angestrebte ACT > 400 s), da es ohne Heparinisierung zu einer Gerinnselbildung im Chemosaturationsfiltersystem kommt [13, 14, 26]. Die Heparinwirkung wird während des gesamten Verfahrens überwacht und ggf. durch wiederholte Dosen an die angestrebte ACT angepasst. Nach dem Ende der Intervention empfiehlt der Hersteller des Filtersystems, die Heparinwirkung mittels Protamin zu antagonisieren, um Blutungskomplikationen zu verhindern [3, 13]. Nach vermehrten Berichten über schwere thrombembolische und allergische Komplikationen verzichtet man in einigen Zentren auf eine generelle Antagonisierung mittels Protamin [15, 22, 23]. Im Rahmen einer Risiko-Nutzen-Abwägung erscheint es empfehlenswert, anhand des individuellen Interventionsverlaufs über die Gabe von Protamin zu entscheiden. Hierzu sind die Bestimmung einer ACT nach Interventionsende und ein interdisziplinärer Konsens zur Gabe von Protamin bzw. zur Indikation von Nachbeatmung noch vor Beendigung der Anästhesie sinnvoll. Im Zentrum der Autoren wird eine ACT < 150 s vor der Verlegung auf die Intensivstation angestrebt.

Ein Großteil der Patienten entwickelt postinterventionell transitorische myeloproliferative Effekte, u. a. in Form einer Leukopenie, Anämie und Thrombopenie [15, 35]. Durch diese Effekte auf das Gerinnungssystem und bei zusätzlichem Heparineffekt können auch im Anschluss der Intervention Blutungskomplikationen auftreten. Daher ist der Kontrolle der Blutgerinnungsparameter und der klinischen Überwachung auf Blutungszeichen eine große Aufmerksamkeit zu schenken. Die Substitution von Blutkomponenten oder Plasmaderivaten sollte faktorenorientiert entsprechend den gültigen Empfehlung der Bundesärztekammer erfolgen [10].

### Hämodynamisches Management

Das hämodynamische Management erfolgt mittels Vasopressorspritzenpumpe (z. B. Noradrenalin) und kristalloider Infusionslösung via *Rapid Infusion System* (RIS). Als Vasopressor wird vom Hersteller primär die kontinuierliche Infusion von im Vergleich zu Noradrenalin etwas weniger potentem Phenylephrin angegeben [13]. In unserem Zentrum haben wir gute Erfahrungen mit Noradrenalin und verwenden es als Standardvasopressor. Aufgrund des vorhersehbaren starken Abfalls des arteriellen Blutdrucks nach der Okklusion des Doppelballonkatheters sollte vor dem Ausklemmen des Bypass ein systolischer Blutdruck von 150–160 mm Hg angestrebt werden [11, 43]. Hier ist im Vorfeld eine besonders klare Kommunikation zwischen allen beteiligten Fachdisziplinen notwendig. Neben der Anpassung der Katecholamindosierung kann dabei auch ein präemptiver Volumenbolus erforderlich sein [3, 11, 43]. Durch den Einsatz großer Infusionsvolumina (ca. 15–30 ml/kgKG*h) kann es in Abhängigkeit vom Ausgangszustand zu einer Dilutionsanämie kommen, die angesichts des eingeschränkten Gerinnungsstatus das Risiko für relevante Blutungseffekte erhöht.

Prinzipiell ist es empfehlenswert, das hämodynamische Management überwiegend durch die Noradrenalindosierung zu steuern, um Überinfusionseffekte (Ödeme, Dilutionseffekte, Senkung der Transfusionsschwelle) zu vermeiden. Im Rahmen des Betriebes des Extrakorporalkreislaufs können als mechanische Komplikationen das temporäre Ansaugen oder die Dislokation des Doppelballonkatheters an der Venenwand auftreten (Absinken der Flussrate). Dabei kann die Volumengabe über die Pumpe und ggf. die Beendigung der Melphalaninfusion erforderlich sein. Daten zu Einsatz und Nutzen eines erweiterten hämodynamischen Monitorings bei PHMP existieren bislang nicht. Die während des Filtereinsatzes häufig beobachtete Notwendigkeit hoher Katecholamindosierungen wurde in der Vergangenheit auch einem Filtrationseffekt von endogenen und exogen zugeführten Katecholaminen zugeschrieben. Beweisende Untersuchungen diesbezüglich sind bislang jedoch nicht publiziert worden [11, 31]. Vor dem Ende der Intervention ist ggf. eine Anpassung der Katecholamindosierung zu empfehlen, um hypertone Entgleisungen und konsekutive Einblutungen bei noch ausgeprägter Antikoagulation zu vermeiden [43].

### Postinterventionelle Phase

Nach einem komplikationslosen Eingriff kann die Allgemeinanästhesie beendet werden und der Patient noch im Angiographiebereich extubiert werden. Postinterventionell sollte routinemäßig eine Überwachung für mindestens einen Tag auf der Intensivstation gewährleistet werden. Bei entsprechenden Kontraindikationen für eine Extubation, wie z. B. ausgeprägten zervikalen Ödemen oder Einblutungen, sollte die Indikation für eine Nachbeatmung auf der Intensivstation großzügig gestellt werden. Unabhängig davon sollten alle erforderlichen Vorkehrungen für eine notfallmäßige Reintubation unter erschwerten Bedingungen sowohl im unmittelbaren Arbeitsbereich der Angiographie als auch auf der Intensivstation getroffen sein [21, 43]. Alle Gefäßkatheter (ZVK und Schleusen) sollten bis zur Normalisierung der Gerinnungsparameter *in situ* verbleiben. Dabei sollten regelmäßige Verlaufskontrollen der Basisgerinnungswerte, aktivierte partielle Thromboplastinzeit (aPTT), Prothrombinzeit (Quick-Wert oder International Normalized Ratio, INR) und Thrombozytenzahl, und arterieller Blutgasanalysen durchgeführt werden. Bei anhaltend eingeschränkter Gerinnungssituation sollte eine gezielte Substitution mit Faktoren bzw. Thrombozyten erwogen werden. Nach der Entfernung der arteriellen Schleuse sollte auf eine ausreichend lange manuelle Kompressionszeit geachtet werden und ein mehrstündiger Druckverband an der Punktionsstelle angelegt werden. Darüber hinaus sollte eine regelmäßige Inspektion der Punktionsstellen hinsichtlich Hämatomen durchgeführt werden. Angesichts häufig beobachteter postinterventioneller passagerer Knochenmarkdepression (Leukopenie, Anämie und Thrombopenie) sowie Leber- und Nierenfunktionseinschränkungen sollten regelmäßige Laborkontrollen erfolgen [43]. Bei insgesamt erhöhtem Risiko für eine postoperative Übelkeit (PONV) sollte eine prophylaktische antiemetische Therapie (z. B. mit Ondansetron) durchgeführt werden. Postinterventionell kann Fieber auftreten, welches symptomatisch mit Antipyretika (z. B. Metamizol oder Ibuprofen) behandelt werden kann. Zur Prophylaxe eines Tumorlysesyndroms wird Allopurinol p.o. für die ersten 3 postoperativen Tage empfohlen. Die Verlegung des Patienten von der Intensivstation kann bei unauffälligem Verlauf in der Regel am Folgetag erfolgen. Die Krankenhausentlassung ist häufig schon nach wenigen Tagen möglich [24, 34, 43].

## Fazit für die Praxis

- Die peri- und postinterventionelle Betreuung von Patienten mit Chemosaturation ist mit ausgeprägten interventionsbedingten hämodynamischen und hämostaseologischen Schwankungen verbunden.
- Ein besonderes Augenmerk erfordert die präoperative Evaluation, um Patienten mit erhöhtem Risiko für Komplikationen zu identifizieren.
- Aus Sicherheitsgründen erfolgt die Anlage der zentralen Venenkatheter ausschließlich ultraschallgestützt.
- Nach Beendigung der Chemosaturation muss eine entsprechende Anpassung der Vasopressordosierung erfolgen, um eine Rebound-Hypertension zu vermeiden.
- Eine klare Kommunikation zwischen Interventionsradiologen, Kardiotechnikern und Anästhesiologen zu den einzelnen Interventionsschritten und insbesondere vor den potenziell kritischen Phasen ist für einen sicheren peri- und postinterventionellen Verlauf essenziell.
